# Post-vaccination incidence and side effects of COVID-19 in a cohort of Brazilian healthcare professionals: an internet-based survey

**DOI:** 10.31744/einstein_journal/2022AO0067

**Published:** 2022-11-09

**Authors:** Matheus Ballestero, Renato Lucas Passos de Souza, Thiago Mamoru Sakae, Luiz Guilherme Villares da Costa, Luciano Furlanetti, Ricardo Santos de Oliveira

**Affiliations:** 1 Universidade Federal de São Carlos São Carlos SP Brazil Universidade Federal de São Carlos, São Carlos, SP, Brazil.; 2 Universidade de São Paulo Faculdade de Medicina de Ribeirão Preto Ribeirão Preto SP Brazil Faculdade de Medicina de Ribeirão Preto, Universidade de São Paulo, Ribeirão Preto, SP, Brazil.; 3 Universidade Federal de Santa Catarina Araranguá SC Brazil Universidade Federal de Santa Catarina, Araranguá, SC, Brazil.; 4 Hospital Israelita Albert Einstein São Paulo SP Brazil Hospital Israelita Albert Einstein, São Paulo, SP, Brazil.; 5 King’s College Hospital Charity London UK King’s College Hospital Charity, London, UK.

**Keywords:** Vaccines, COVID-19, Coronavirus infections, CoronaVac, Health personnel, Drug-related side effects and adverse reactions

## Abstract

**Objective:**

So far, at least 18 different severe acute respiratory syndrome coronavirus-2 vaccines have been approved. Until October 2022, 12.8 billion doses had been administered all over the world. Vaccination of high-risk groups and healthcare professionals was initially prioritized. This cross-sectional survey aimed to investigate the occurrence of vaccine side effects, as well as the incidence of COVID-19 among vaccinated healthcare professionals.

**Methods:**

A survey was structured and shared with healthcare professionals using a digital platform to collect data between May and June 2021.

**Results:**

This study included 6,115 participants. The most prevalent age group was 30-39 years (31.3%), 67.3% were female and 73.2% accounted for physicians, and nearly half worked in frontline care for COVID-19. Approximately, two-thirds of them were vaccinated with CoronaVac, and about 60% reported at least one side effect following the vaccination. Nevertheless, minor reactions were more frequent, such as pain at site of injection, fatigue, and headache. Our data could be used to inform people on the likelihood of side effects of COVID-19 vaccines, particularly CoronaVac, since this is the largest study about vaccine reactions using this vaccine, to our best knowledge.

**Conclusion:**

The incidence of side effects in Brazilian healthcare professionals was 60%, and the most common side effects included local swelling/pain, fatigue/tiredness, fever, headache, and limb pain.

## INTRODUCTION

Since the first reports in Wuhan (Hubei province, China) on December 31, 2019, coronavirus disease 2019 (COVID-19) has posed unprecedented challenges to the world.^(1)^ The severe acute respiratory syndrome coronavirus 2 (SARS-CoV-2) was identified as the pathogenic agent of COVID-19. Until June 2022, the virus infected over 547 million individuals and caused more than 6.3 million deaths worldwide.^(2,3)^

On December 2^nd^, 2020, the United Kingdom gave temporary regulatory approval for the Pfizer/BioNTech vaccine, and was the first region to approve COVID-19 vaccination in the Western world. On December 31, 2020, the World Health Organization (WHO) listed the BNT162b2 COVID-19 mRNA vaccine for emergency use, making the Pfizer/BioNTech vaccine the first to receive emergency validation.^(4)^ Concomitant with the further development of vaccines, the WHO has strongly recommended intensive vaccination programs all over the world.

Since then, at least 18 different vaccines have been approved and until October 2022, 12.8 billion doses were administered throughout the world.^(5)^ Vaccination strategies varied in all continents. Due to the increased viral exposure at the workplace and higher risk of developing severe diseases, healthcare professionals have been given priority. In Brazil, four types of vaccine (CoronaVac-Butantan/Sinovac; ChAdOx1-Oxford/Astrazeneca; Ad26.COV2 S-Janssen; and BNT162b2/Pfizer/BioNTech) received governmental approval and registration.^(3,6)^

Several adverse reactions due to vaccination have been reported. Both active component (antigen) and other vaccine components (adjuvants) can cause hypersensitivity and anaphylaxis, which is the most severe hypersensitivity reaction. The incidence rate of anaphylaxis before COVID-19 was approximately 1.31 per million vaccine doses.^(7)^ The proinflammatory cytokines interleukin 1 and 6 and tumor necrosis factor α are released in response to vaccination, causing pain at the injection site by inducing inflammation. Additionally, they may cause systemic symptoms, such as headache, fatigue, malaise, nausea, and fever, which are similar to symptoms of some infectious diseases, including COVID-19.^(8)^

Most of the major vaccine reactions are compulsorily notified; however, minor reactions, such as headache and myalgia, are usually underreported.^(3,6,9)^

To the best of my knowledge, this is the largest study published about adverse effects of CoronaVac-Butantan/Sinovac vaccine.

## OBJECTIVE

To investigate the occurrence of vaccine side effects, as well as the incidence of COVID-19 among vaccinated healthcare professionals.

## METHODS

### Study design

This was a cross-sectional study with data gathered by means of an internet survey. The questionnaire was structured and shared using the digital platform Google Forms™.

The survey respondents were invited to take part in the study through different online platforms. The survey link was promoted through e-mail communications and social media (Facebook™, e-mail and WhatsApp™). The questionnaire was designed in a user-friendly layout, with clear answering instructions that required only minimal computer/smartphone skills to navigate and complete. The survey link was shared on May 16, 2020, and the responses were collected until June 7, 2021. Participation was voluntary and included informed consent and anonymity; hence, no personal information was collected, and respondents could not be identified.

At the time of survey, the Brazilian government adopted massive vaccination of health professionals with multiple vaccines available, mainly CoronaVac. The questionnaire was self-administered in Portuguese and is described in appendix 1.

### Data analysis

The online response submitted by respondents was transferred to an Microsoft Excel database and exported to SPSS version 21.0 for analysis. Fisher´s exact test was used to compare means of selected background variables. Bivariate and multivariate logistic regression analyses were used to compare the groups and to predict the relations between dependent and independent variables. A 95% confidence interval (95%CI) and a p<0.05 were used to determine a statistically significant association.

### Ethical aspects

This study was submitted to and approved by the Research Ethics Committee of *Hospital das Clínicas, Faculdade de Medicina de Ribeirão Preto, Universidade de São Paulo* (FMRP USP), Ribeirão Preto, SP, Brazil, (CAAE: 43104821.2.0000.5440; #4.538.515). The patients included in the study signed an informed consent term. This study was conducted according to the Declaration of Helsinki.

## RESULTS

A total of 6,115 healthcare professionals responded to the online questionnaire and were included in the study; no response was excluded. The most prevalent age group was between 30-39 years (31.3%), with a predominance of females (67.3%).

The sample comprised 4,472 (73.2%) physicians, 446 (7.6%) nurses, 206 (3.4%) physical therapists, and the remaining approximately 15% accounted for other professionals (Table 1).

**Table 1:** Sociodemographic characteristics of the cohort

Variable		n (%)
Sex		
	Male	1,992 (32.6)
	Female	4,117 (67.3)
	Other	378 (6.2)
Age group		
	20-29 years	1,371 (22.4)
	30-39 years	1,916 (31.3)
	40-49 years	1,300 (21.3)
	50-59 years	892 (14.6)
	60-69 years	514 (8.4)
	70-79 years	115 (1.9)
	>80 years	7 (0.1)
Occupation		
	Physician	4,472 (73.2)
	Nurse	466 (7.6)
	Physical therapist	206 (3.4)
	Occupational therapist	11 (0.2)
	Pharmacist	95 (1.6)
	Speech therapist	19 (0.3)
	Physical educator	9 (0.1)
	Psychologist	167 (2.7)
	Dietitian	39 (0.6)
	Social worker	14 (0.2)
	Laboratory technician	5 (0.1)
	Dental surgeon	206 (3.4)
	Oral hygiene technician	7 (0.1)
	Biomedical professional	21 (0.3)
Work in the frontline of COVID-19		
	Yes	3,116 (51)
	No	2,999 (49)
Brazilian region		
	Midwest	259 (4.2)
	Federal District	255 (4.2)
	North	204 (3.3)
	Northeast	918 (15)

A total of 3,116 (51%) participants had worked directly with COVID-19 patients (*e.g.,* in accident and emergency - departments, or intensive care units). Almost one third of them (30.5%) reported being diagnosed as COVID-19 before the vaccination, whereas 1.8% developed reinfection despite immunization (Table 2).

**Table 2:** COVID-19 characteristics

Variable		n (%)
Had COVID-19?		
	Yes	1,864 (30.5)
	No	4,254 (69.9)
Had reinfection?		
	Yes	111 (1.8)
	No	6,004 (98.2)
When was your first dose?		
	February 2021	3,631 (59.4)
	Before January 17, 2021	238 (3.9)
	Between January 18 and 24, 2021	1,265 (20.7)
	Between January 25 and 31, 2021	962 (15.7)
	Not vaccinated	19 (0.3)

The incidence of post-vaccine infection (professionals previously vaccinated who were infected with COVID-19) was 4.9%, whereas 5.7% received CoronaVac and 3.3% ChAdOx1.

CoronaVac was administered to 3,911 (64%) individuals, ChAdOx1 to 2,091 (34.2%), BNT162b2 to 68 (1.1%), Ad26.COV2.S to 9 (0.1%), Gam-COVID-Vac to 7 (0.1%), and Moderna to one. The second dose was given to 86.4% of participants when responding to the survey.

Comorbidities were recorded in 1,481 participants (24.2%), and hypertension was the most prevalent (11.2%), followed by *diabetes mellitus* (3.8%), allergies (4.7%) and lung disease (3.1%). Approximately two thirds of participants were vaccinated with CoronaVac (Table 3).

**Table 3:** Clinical characteristics of sample

Variable		n (%)
Has comorbidities?		
	Yes	1,481 (24.2)
	No	4,634 (75.8)
Which comorbidities?		
	Hypertension	682 (11.2)
	Diabetes mellitus	234 (3.8)
	Lung disease	188 (3.1)
	Cardiovascular disease	113 (1.8)
	Immunodeficiency	30 (0.5)
	Allergies	290 (4.7)
	Others	512 (8.4)
	No	4,569 (74.4)
What type of vaccine did you receive?		
	Pfizer/BioNTech - BNT162b2	68 (1.1)
	Moderna	1 (0.0)
	Oxford/AstraZeneca - ChAdOx1	2,091 (34.2)
	Sputnik V - Gam-COVID-Vac	7 (0.1)
	Sinovac - CoronaVac	3,911 (64)
	Janssen/Johnson - Ad26.COV2.S	9 (0.1)
	Other	22 (0.4)
Did you experience any of these side effects after first dose?		
	Local swelling/pain	2,129 (34.8)
	Fatigue/tiredness	1,316 (21.5)
	Fever	854 (14)
	Headache	1,380 (22.6)
	Limb pain	1,007 (16.5)
	Nausea	35 (0.5)
	Cough	111 (1.8)
	Diarrhea	26 (0.4)
	Chills	23 (0.4)
	Itching	11 (0.2)
	Facial paralysis	3 (0.0)
	Myelitis/Guillain Barre	2 (0.0)
	Anosmia	23 (0.4)
	Herpes zoster	8 (0.1)
	No symptoms	2,503 (40.9)
Time to present signs and symptoms after the first dose		
	Until 3 days	2,926 (47.8)
	4-7 days	97 (1.6)
	>8 days	62 (1)
	No	3,031 (49.6)
How long after the first dose did signs and symptoms regress?	
	Up to 24 hours	1,011 (16.5)
	Up to 48 hours	1,088 (17.6)
	Up to 72 hours	568 (9.3)
	>72 hours	430 (7)
	Had no symptoms	3,019 (49.4)
Did signs and symptoms regress spontaneously after the first dose?		
	Yes	2,882 (47.1)
	No	216 (3.5)
	Had no symptoms	3,013 (49.3)
Symptoms after first dose		
	Yes	3,188 (52.1)
	No	2,927 (47.9)
Symptoms after the second dose		
	Yes	2,421 (39.6)
	No	2,860 (46.8)
	Did not receive the second dose	834 (13.6)
Symptoms after some dose		
	Yes	4,020 (65.7)
	No	2,095 (34.3)
Have you been given 2 doses?		
	Yes	5,114 (83.7)
	No	999 (16.3)
Time to present signs and symptoms after the second dose		
	Up to 3 days	1,744 (28.5)
	4-8 days	87 (1.4)
	>8 days	47 (0.8)
	No	3,206 (52.4)
How long after the second dose did signs and symptoms regress?		
	Up to 24 hours	754 (12.3)
	Up to 48 hours	600 (9.8)
	Up to 72 hours	283 (4.6)
	>72 hours	241 (3.9)
	Did not receive two doses	995 (16.3)
	No side effects	3,240 (53)
Did signs and symptoms regress spontaneously at the second dose?		
	Yes	1,777 (16.4)
	No	108 (1.8)
Did you get COVID-19 after the vaccine?		
	Yes	297 (4.9)
	No	5,817 (95.1)
If you were infected after the vaccine and answered “Yes “ to the above question, were you hospitalized?		
	Yes	15 (5)
	No	284 (95)

About 60% had symptoms after the vaccine and the most prevalent side effects were pain at site of injection (34.8%), fatigue (21.5%) and headache (22.6%). Approximately, half had the symptoms until three days after vaccination, whereas 3,188 (52.1%) had symptoms after the first vaccine dose, and 2,412 (39.6%) had the symptoms after the second dose. Additionally, 834 (13.6%) participants did not take the second dose at the time of the survey (Table 3).

### Bivariate analysis

Professionals who received CoronaVac had a risk of post-vaccination infection approximately 80% higher (risk ratio [RR] = 1.78; 95%CI: 1.37-2.31; p<0.0001). No statistically significant differences were found regarding type of vaccine and admission to hospital (p=0.257).

The age group below 40 years had a 30% higher risk of post-vaccination symptoms (RR=1.32; 95%CI: 1.27-1.37; p<0.0001). Age groups under 40 years had a greater chance of suffering from COVID-19 before vaccination (RR=1.23; 95%CI: 1.14-1.33; p<0.0001). The age group below 40 years had a greater chance of having reinfection (RR=1.65; 95%CI: 1.12-2.43; p<0.0001). Age group was not associated with COVID-19 after vaccination (p=0.592).

Female health professionals had a 50% higher incidence of post-vaccination signs and symptoms (RR=1.51; 95%CI: 1.42-1.61; p<0.0001). Males had a 9% higher incidence of COVID-19 before the vaccine (RR=1.09; 95%CI: 1.01-1.18; p=0.022). Males had a six times greater risk of hospitalization after vaccination (RR=6.49; 95%CI: 1.87-22.51; p<0.0001). No differences were found in sex and reinfection (p=0.17) or post-vaccine COVID-19 (p=0.083).

### Multivariate analysis

In the multivariate logistic regression analysis, use of CoronaVac vaccine, male sex, age under 40 years and having comorbidities use of CoronaVac vaccine, male sex, age under 40 years and having comorbidites were independently associated with developing post-vaccination symptoms. CoronaVac vaccine was the strongest factor associated with post-vaccination symptoms followed by male sex, both with more than double odds. On the other hand, working on the COVID-19 frontline, reinfection and having COVID-19 post-vaccination were factors not associated with post-vaccination symptoms. (Table 4)

**Table 4:** Multivariate logistic regression analysis of factors associated with the development of post-vaccination symptoms in healthcare workers

Variable	OR (adjusted)	95%CI	p value
Vaccination with Coronavac	5.98	5.16-6.94	<0.001*
Male gender	2.37	2.10-2.68	<0.001*
Age<40 years old	2.20	1.94-2.49	<0.001*
Work with Covid-19	1.06	0.93-1.20	0.37
Had COVID-19 disease	1.11	0.97-1.27	0.13
Had Reinfection	0.96	0.61-1.52	0.86
Comorbidities	1.26	1.09-1.45	0.001*
Had COVID-19 post vaccination	0.79	0.60-1.05	0.11

* p<0.05 (statistically significant).

95%CI: 95% confidence interval.

## DISCUSSION

Since the start of vaccine production, people have expressed worries about hazards and risks of getting vaccined, often caused by misinformation. These movements led the WHO to consider vaccine hesitancy as one of top ten threats to global health.^(10)^ Currently, massive vaccination is the main strategy to control the COVID-19 pandemic and is supported by the WHO^(11)^ and the United States Food and Drug Administration (FDA).^(12)^

Vaccine efficacy varies; however, it reduces mortality and cases of severe disease depending on the type of vaccine and characteristics of the vaccinated population.^(13)^ Vaccine effectiveness is the percentage reduction of disease cases in a vaccinated group of people compared to an unvaccinated group. COVID-19 efficacy is measured by the development of disease or severe illness and death by COVID-19.^(14)^

The efficacy of CoronaVac (administered in 64% of participants) in a large phase III trial in Brazil showed that two doses, at an interval of 14 days, had an efficacy of 51% against symptomatic COVID-19 infection, 100% against severe COVID-19, and 100% against hospitalization, starting 14 days after the second dose.^(15)^ Another phase III clinical trial published showed efficacy to prevent COVID-19 infection using BNT162b2 of 95%,^(16)^ Moderna of 94.1%,^(17)^ ChAdOx1 of 70.40%^(18)^ and Gam-COVID-Vac of 91.6%.^(18,19)^

The present retrospective survey and bivariate analysis revealed professionals who received CoronaVac had an approximately 80% higher risk of post-vaccination infection (RR=1.78; 95%CI: 1.37-2.31; p<0.0001), and only 15 (5%) were admitted to hospital. The age group was not associated with suffering from COVID-19 after vaccine (p=0.592).

Data about COVID-19 vaccine adverse effects are heterogeneous due to vaccine diversity. Menni et al.,^(20)^ in a prospective observational study, followed up 627,383 individuals immunized with BNT162b2 and ChAdOx1 COVID-19 vaccines. Among those vaccinated, 159,101 (25.4%) reported having one or more systemic adverse effects, whereas 257,209 (66.2%) of 388,430 reported one or more local adverse effects. The most commonly reported systemic side effects included fatigue and headache, within the first 24 hours after vaccination and lasted a mean of 1.01 days. Tenderness and pain in the injection site were the most frequently reported local effects occurring on the day after injection and lasting a mean of 1.02 days. The comparison of systemic effects after one dose of each vaccine revealed significantly higher reactogenicity in individuals who had one dose of the ChAdOx1 vaccine than in those who had one dose of the BNT162b2 vaccine (p<0.0001). Other side effects included allergic skin reactions, such as skin burning, rashes and red welts on the lips and face, and were reported by 10,860 (1.7%) out of 627,383 users regarding both types of vaccine, but none was severe.

The minor to moderate side effects of Pfizer-BioNTech vaccine in 455 Saudi Arabian inhabitants, in a retrospective online survey, revealed 98.4% complained of symptoms, mostly injection site pain, headache, flu-like symptoms, fever and fatigue.^(21)^

Our study included 3,911 (64%) health professionals who were vaccinated with CoronaVac and, to our best knowledge, this is the largest phase IV study on adverse events of CoronaVac.

Our study found approximately 60% of adverse effects, whereas Kaya et al., who conducted a prospective study with 329 health professionals vaccinated with CoronaVac in Turkey, found adverse effects in only 33.2% of participants (including pain, redness, swelling, headache fever, state of sleep/fatigue, nausea/vomiting, allergy, extensive pruritus and myalgia). The three most common severe adverse reactions were headache, state of sleep/fatigue, pain and redness.^(22)^ Another study from Turkey with 780 healthcare workers (cross-sectional study) showed 62.5% of professionals experienced at least one adverse effect, and injection site pain was the most frequent (41.5%), and fatigue (23.6%), headache (18.7%), muscle pain (11.2%) and joint pain (5.9%) were the common systemic adverse effects. Female workers were significantly more often affected (67.9%).^(23)^

## CONCLUSION

The incidence of side effects in our sample of vaccinated Brazilian healthcare professionals was high, and the most common side effects were local swelling/pain, fatigue/tiredness, fever, headache, and limb pain. The incidence of side effects with CoronaVac was much lower than in previous publications with this vaccine, probably due to the short time between the vaccine and the survey, but higher than with other vaccines. Our data could be used to inform people on the likelihood of side effects of COVID-19 vaccines, particularly CoronaVac, since this is the largest study with vaccine reaction using this vaccine, to our best knowledge.

## Appendix 1 Questionnaire applied in the online surgey

**Table:**

State of the federation where you work – Acre – Alagoas – Amapá – Amazonas – Bahia – Ceará – Federal District – Espírito Santo – Goiás – Maranhão – Mato Grosso – Mato Grosso do Sul – Minas Gerais – Paraíba – Paraná – Pernambuco – Piauí – Rio de Janeiro – Rio Grande do Norte – Rio Grande do Sul – Rondônia – Roraima – Santa Catarina – São Paulo – Sergipe – Tocantins Area of expertise? – Nursing – Medicine – Physiotherapy – Pharmacy – Speech Therapy – Occupational Therapy – Physical Education – Psychology – Nutrition – Social Work – Laboratory Technician – Dentistry – Assistant Dental Office Technician – Oral Hygiene Biomedicine – Other Sex – Male – Female – Other Age group? – 20-29 years – 30-39 years – 40-49 years – 50-59 years – 60-69 years – 70-79 years – Over 80 years Do you work, or have you worked on the COVID-19 frontline? – Yes – No Have you had COVID-19? – Yes – No Have you had reinfection (new positive PCR test after cure of COVID-19)? – Yes – No When did you receive the first (or only) dose of the vaccine? – Before January 17, 2021 – Between January 18 and 24, 2021 – Between January 25 and 31, 2021 – As from February 2021 – I have not been vaccinated Do you have any comorbidities? – Yes – No Type of comorbidity – Hypertension – *Diabetes mellitus* – Lung disease – Heart disease – Immunodeficiency – Allergy – Others – I have no comorbid diseases Which vaccine did you receive? – Biotech-Pfizer – Modern – Oxford - AstraZeneca – Sputnik V – CoronaVac – Janssen – Other Did you experience any of these effects after the first dose of the vaccine? – Swelling or pain at the site of injection – Local redness – Fatigue/tiredness – Fever – Headache – Pain in upper and/or lower limbs – Cough – Itching – Shock (allergic) – Facial paralysis – Myelitis/Guillain Barre – Anosmia – Herpes zoster – I had no post-vaccine signs and symptoms – Other Time from vaccination to onset of signs and symptoms after the first dose of vaccine – Up to 3 days – 4-7 days – Over 8 days – No post-vaccine signs and symptoms How long after the first dose did post-vaccine signs and symptoms regress? – 24 hours – Up to 48 hours – Up to 72 hours – More than 72 hours – I had no post-vaccine signs and symptom Did signs and symptoms regress spontaneously after the first dose? – Yes – No – I had no side effects from the vaccine Have you received the second dose of the vaccine? – Yes – No Did you experience any of these effects after the second dose of the vaccine? – Swelling or pain at site of injection / local redness – Fatigue / tiredness – Fever – Headache – Pain in upper and/or lower limbs – Cough – Itching – Shock (allergic) – Facial paralysis – Myelitis/Guillain Barre – Anosmia – I had no post-vaccine signs and symptoms – Herpes zoster – Had no side effects – I was not given the second dose – Other: Time from vaccination to onset of signs and symptoms after the second dose of vaccine – Up to 3 days – 4-7 days – Over 8 days – No post-vaccine signs and symptoms – I have not received the second dose of the vaccine How long after the second dose did post-vaccine signs and symptoms regress? – 24 hours – Up to 48 hours – Up to 72 hours – More than 72 hours – I had no post-vaccine effect – I have not received the second dose Did signs and symptoms regress spontaneously after the second dose? – Yes – No – I had no side effects from the vaccine – I have not received the second dose Did you get COVID-19 after the vaccine? – Yes – No If you were infected after the vaccine and answered “Yes” to the above question, were you hospitalized? – Yes – No
